# Group and Individual Variability in Mouse Pup Isolation Calls Recorded on the Same Day Show Stability

**DOI:** 10.3389/fnbeh.2017.00243

**Published:** 2017-12-15

**Authors:** Terra D. Barnes, Michael A. Rieger, Joseph D. Dougherty, Timothy E. Holy

**Affiliations:** ^1^Department of Neuroscience, School of Medicine, Washington University in St. Louis, St. Louis, MO, United States; ^2^Department of Genetics and Department of Psychiatry, School of Medicine, Washington University in St. Louis, St. Louis, MO, United States

**Keywords:** mouse vocalizations, pup-isolation calls, ultrasonic vocalizations, reproducibility, variability

## Abstract

Mice produce ultrasonic vocalizations (USVs) in a variety of social situations, and USVs have been leveraged to study many neurological diseases including verbal dyspraxia, depression, autism and stuttering. Pups produce isolation calls, a common USV, spontaneously when they are isolated from their mother during the first 2 weeks of life. Several genetic manipulations affect (and often reduce) pup isolation calls in mice. To facilitate the use of this assay as a means of testing whether significant functional differences in genotypes exist instead of contextual differences, we test the variability inherent in many commons measures of mouse vocalizations. Here we use biological consistency as a way of determining which are reproducible in mouse pup vocalizations. We present a comprehensive analysis of the normal variability of these vocalizations in groups of mice, individual mice and different strains of mice. To control for maturation effects, we recorded pup isolation calls in the same group of C57BL/6J 5 days old mice twice, with 1 h of rest in between recordings. In almost all cases, the group averages between the first and second recordings were the same. We also found that there were high correlations in some parameters in individual mice across recording while others were not well correlated. These findings could be replicated for the majority of features in a separate group of C57BL/6J mice and a group of 129/SvEvBrd-C57BL/6J mice. The averages of these mouse USV features are highly consistent and represent a robust assay to test the effects of genetic and other interventions in the experimental setting.

## Introduction

Mouse ultrasonic vocalizations (USVs) are a recognized assay of neurological function in mice and reflect the animal’s cognitive and social state (Branchi et al., 2001; Hofer et al., 2002; Fisher et al., 2003; Liu et al., 2003; Scattoni et al., 2009; Hammerschmidt et al., 2012). Mouse USVs have been leveraged to study many neurological diseases including verbal dyspraxia, depression, autism and stuttering (Fisher et al., 2003; Shu et al., 2005; Fujita et al., 2008; Enard et al., 2009; Fischer and Hammerschmidt, 2010; Gaub et al., 2010; Scattoni et al., 2011; Ey et al., 2013; Barnes et al., 2016). Pup isolation calls are a common type of USV that are spontaneously produced when pups are isolated from their mother during the first 2 weeks of life. Pup isolation calls are affected by a wide variety of genetic manipulations (Nastiti et al., 1991; Nelson and Panksepp, 1998; Weller et al., 2003; Antonelli et al., 2005; Takayanagi et al., 2005; Scattoni et al., 2008) that often decrease the vocalization number (Nastiti et al., 1991; Nelson and Panksepp, 1998; Branchi et al., 2001; Fischer and Hammerschmidt, 2010; Gaub et al., 2010).

One disorder that has been extensively studied utilizing mouse USVs is speech apraxia caused by a heterozygous mutation in *FOXP2*. However the data resulting from pup isolation call assays do not always agree. Shu et al. (2005) found that mice heterozygous for a knockout of Foxp2 had a lower call rate compared to wild type littermates. In contrast Gaub et al. (2010) showed there was no effect in mice heterozygous for the Foxp2 S321X nonsense mutation that representing a null allele. Another disorder that has been explored utilizing USVs in mutant mice is autism. *ProSAP1/Shank2*^−/−^ mouse pups have been found to have a similar call rate compared to wildtype mice on P6 or a significantly different call rate depending on the study (Schmeisser et al., 2012; Ey et al., 2013).

We therefore wanted to characterize the variability inherent in these types of measurements. Despite the widespread use of USVs to study inter-group differences, few studies have examined the stability of features (such as call rate) in the same group of mice. Understanding and recognizing natural variability is crucial when interpreting many USV features including call rate, pitch jump and power over several days (i.e., during critical early maturation) but that most USV features were consistent within a single recording session. They proposed that many USV features are strongly influenced by “state”-like variables (Rieger and Dougherty, 2016).

Maturation also clearly affects many mouse pup isolation call features; for example, vocalization duration decreases and bout complexity increases as pups age (Hahn et al., 1998; Liu et al., 2003; Grimsley et al., 2011; Rieger and Dougherty, 2016). In addition, mice become less repetitive in their syllable choices over time (Grimsley et al., 2011). Mouly et al examined maturation effects on the USVs elicited by a mild foot shock. In infant juvenile and adult rats, mild foot shock elicited a single class of USVs and found that mild foot shock elicited a single class of USV that changed in frequency and duration based on age (Boulanger-Bertolus et al., 2017).

We therefore sought to characterize vocalization feature stability in the same mouse pups while controlling for maturation effects. We determined vocalization parameter consistency for a given mouse and for groups of mice across two identical experimental replicates separated by 1 h of rest in the home cage. To elicit pup isolation calls, pups were separated from the dam, recorded, and then returned to the dam. Recordings were compared to determine mouse vocalization feature variability in groups of animals as well as in individual animals. We show that, across strains and replicates, group averages of all the tested features were stable and reproducible across recording time points. In contrast, some features showed variability in individual mice recorded 1 h apart. Nevertheless, the averages of these mouse vocalization features are generally consistent, and recording USVs in mouse pups represents a robust assay to test the effects of genetic and other interventions in the experimental setting.

## Materials and Methods

### Animals and Experimental Protocol

C57BL/6J mice were acquired from The Jackson Laboratory (Bar Harbor, ME) and bred locally to produce experimental cohort for cohorts 1 and 2. A mixed genetic background was produced with 129/SvEvBrd and C57BL/6J crosses for cohort 3. Mice were kept in a 12 h light-dark cycle and were tested during the light part of the cycle. Mice received standard chow and water *ad libitum*. Dams were checked for pups daily, and the first day that a litter was discovered was considered postnatal day zero.

Litters were excluded if they did not have at least four pups. Mice from cohort 1 came from five separate litters with an average size of 7.2 (range 4–9 pups). Mice from cohort 2 came from three separate litters with an average size of 7.09 (range 6–8 pups). Finally mice from cohort 3 came from three separate litters with an average size of 8.6 (range 7–10 pups).

The testing equipment was as described previously (Barnes et al., 2016). Recordings occurred in a wooden enclosure (to attenuate external sounds) measuring 33.1 × 20.3 × 16.8 cm with a transparent Plexiglas front. Sounds were digitized at 250 kHz at 16-bit resolution (National Instruments, Austin, TX, USA). The microphone (from Avisoft Bioacoustics) was suspended from the top of the cage approximately 5 cm from the bottom of the recording box. Mice that did not have at least 10 calls in both recording sessions were excluded from the analysis.

Mice were recorded on postnatal day 5 (P5). We chose P5 as mice pups vocalize sufficiently frequently by this time. In addition, although, maternal potentiation has been found in mouse pups at P8–P12 (Moles et al., 2004; Scattoni et al., 2008; Young et al., 2010), it has not been documented earlier. For recordings, the dam was removed from the cage and placed in a new clean cage away from the home cage. The home cage containing pups was placed in an incubator at 34°C. Before testing, the auxiliary temperature of the pups was taken with a flexible thermistor on the back of the animal (Omega Engineering Ltd., Manchester, UK). Ten minutes after the dam’s removal from the home cage, the first pup was placed into the test chamber. Recordings lasted 5 min. Afterwards, the pup was weighed and marked on the back with an odorless ink to allow for subsequent identification. The pup was then returned to the dam (Hofer et al., 2002) and the next pup placed into the recording chamber. After all pups had been recorded once, the dam and pups were returned to their original cage and placed back in their original location on the housing shelf. After an hour, the cage was taken back into the recording room and the entire procedure repeated as per the first run with the pups recorded in the same order as in the first set of recordings.

### Statistical Analysis

All analyses were performed using in-house MATLAB (MathWorks, Natick, MA, USA) code, some of which are available online at http://holylab.wustl.edu/. Waveforms were pre-processed, band-pass filtered (25–110 kHz) and calls identified using mean frequency, “spectral purity” (fraction of total power concentrated into a single frequency bin) and the “spectral discontinuity” (the change in the allocation of power across frequencies between two adjacent time bins) as previously described (Barnes et al., 2016). Stored acoustical waveforms were processed using MATLAB to compute the sonogram (512 samples/block, half-overlap, resulting in a time resolution of 1.02 ms and a frequency resolution of 0.49 kHz).

The analysis code implemented fully-automated algorithms and was therefore blind to mouse strain, recording session, or cohort. To calculate the number of calls and the duration of calls and pauses, each vocalization or pause contributed to the mean for each animal or subject; each individual’s mean was then averaged to obtain the group mean. A within-subject paired *t*-test was then performed to compare groups with each individual’s mean.

Bout-level analyses defined bouts based on histograms of pause lengths for all groups of mice. Histograms were constructed with a range of bin sizes (0.05–0.3 s). The middle of the bin with fewest counts in the range of 0.15–0.33 s was averaged across all bin sizes to determine the criterion for an inter-bout pause. The resulting intra-bout/inter-bout cutoff was 0.322 s. The hyper-bout/intra-bout cutoff was determined in the same way with the range of 0.05–0.1 s. The resulting hyper-bout/intra-bout cutoff was 0.094. For the replication experiment, the resulting intra-bout/inter-bout cutoff was 0.298 s and the hyper-bout/intra-bout cutoff was 0.083. For the 129/SvEvBrd-C57BL/6J) pups, the intra-bout/inter-bout cutoff was 0.250 s and the hyper-bout/intra-bout cutoff was 0.072.

For duration, we calculated the mean of all vocalizations in an individual mouse’s recording session for times *t* and *t* + 1. A paired *t*-test was then used to determine whether there was a significant difference across the population. The same procedure was used for all other parameters. For calculations involving power, the mean power of each vocalization was first calculated and then averaged over the session for each animal. To calculate the hyper-pause duration, one animal was excluded as it did not have any pauses that short.

The variance of each parameter for a single recording session was divided by the mean. Data were bootstrapped 10,000 times to calculate confidence intervals (CI) that were used to determine significance.

We used a leave-one-out strategy for the linear discriminant analysis (LDA) and *k*-nearest neighbor analysis, excluding each data point consecutively and classifying it as having been recorded at time *t* or *t* + 1 based on the identity of the three closest data points. When attempting to classify the recordings based on the individual, only the nearest neighbor was used.

## Results

**Figure 1:**
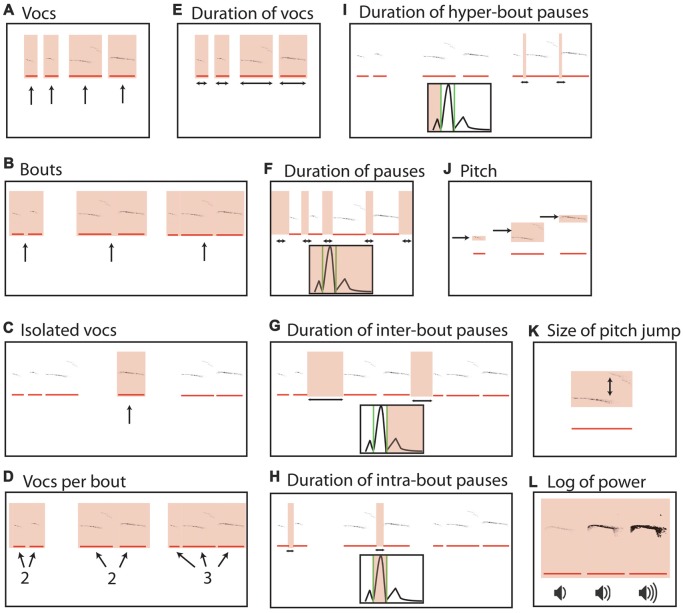
Parameters of pup isolation calls. **(A)** Mean vocalization number in each recording. Gray bars indicate recordings taken after 1 h of rest (*t* + 1 h). Error bars are the standard error of the mean. **(B)** Number of bouts per recording. **(C)** Percentage of isolated vocalizations. **(D)** Vocalizations per bout. **(E)** Mean duration of vocalizations. **(F)** Mean duration of pauses between vocalizations. **(G)** Mean pause duration of inter-bout vocalization. **(H)** Mean pause duration of intra-bout vocalization. **(I)** Mean pause duration of hyper-bout vocalization. **(J)** Mean frequency of vocalizations. **(K)** Mean pitch jump of vocalization. **(L)** Mean log power of vocalizations. All examples are representative depictions and are not real data.

Isolation calls were recorded in 35 C57BL/6J P5 pups (cohort 1). Each pup was recorded for 5 min before being returned to their home cages on their home rack for 1 h. The same pups were then recorded again in the same order. USVs were then analyzed to determine the consistency of 12 USV parameters (Figure 1) at the group and individual level across the two time points. Eleven pups were excluded for not having at least 20 calls in both recordings and two were excluded for noise contamination, leaving 22 recording pairs for analysis. Mice weighed an average 2.27 ± 0.20 g. The mean temperature of the mice directly before recording at time *t* and *t* + 1 were not significantly different (32.2 ± 0.3° and 31.5 ± 0.3°).

### Group-level Consistency between Two Recordings Taken 1 h Apart

**Figure 2:**
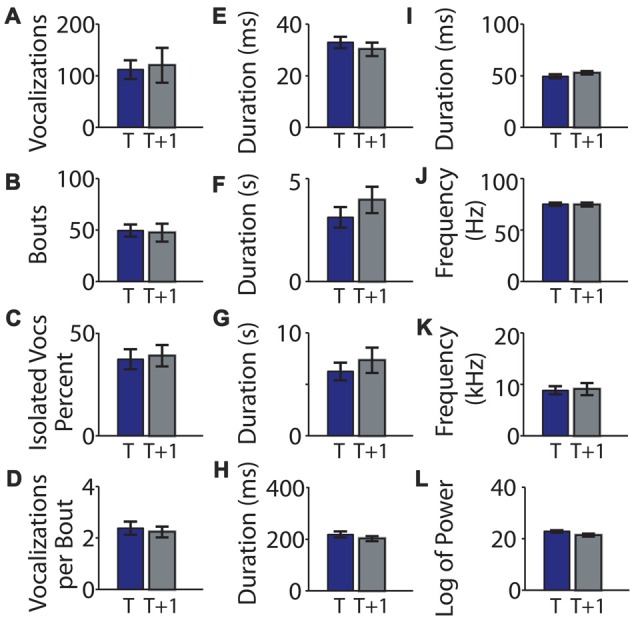
Means of recorded mouse vocalization parameters separated by an hour of rest are consistent. **(A)** Mean vocalization number in each recording. Blue bars indicate the first recording taken on post-natal day 5 (*t*). Gray bars indicate recordings taken after 1 h of rest (*t* + 1 h). Error bars are the standard error of the mean. **(B)** Number of bouts per recording. **(C)** Percentage of isolated vocalizations. **(D)** Vocalizations per bout. **(E)** Mean duration of vocalizations. **(F)** Mean duration of pauses between vocalizations. **(G)** Mean pause duration of inter-bout vocalization. **(H)** Mean pause duration of intra-bout vocalization. **(I)** Mean pause duration of hyper-bout vocalization. **(J)** Mean frequency of vocalizations. **(K)** Mean pitch jump of vocalization. **(L)** Mean log power of vocalizations. No significant differences were found.

The mean number of vocalizations in the first recording at time *t* and the second recording at *t* + 1 was not significantly different (111.864 ± 18.263 vs. 120.500 ± 33.858; paired *t*-test, *t* = −0.29, *p* = 0.78, Figure 2A). Likewise, the number of bouts per recording (49.409 ± 5.865 and 47.273 ± 8.798; *t* = 0.22, *p* = 0.83, Figure 2B), the mean number of isolated vocalizations (0.371 ± 0.049 and 0.388 ± 0.052; *t* = −0.37, *p* = 0.71, Figure 2C) and mean vocalizations per bout (2.374 ± 0.252 and 2.227 ± 0.214; *t* = 0.61, *p* = 0.55, Figure 2D) were not significantly different between time points.

We next compared vocalization duration and pauses at each time point. The mean vocalization duration (0.033 ± 0.002 and 0.030 ± 0.003; *t* = 0.96, *p* = 0.35, Figure 2E) and mean duration of pauses between vocalizations (3.106 ± 0.497 and 3.955 ± 0.639; *t* = −1.04, *p* = 0.31, Figure 2F) were not significantly different between the two time points. We next divided the pauses into: (i) pauses between bouts of vocalizations (inter-bout pauses); (ii) long pauses between vocalizations inside a bout (intra-bout pauses); and (iii) quick short pauses between vocalizations inside a bout (hyper-bout pauses; Ey et al., 2013; Barnes et al., 2016). The mean inter-bout pause duration (6.235 ± 0.854 and 7.319 ± 1.237; *t* = −0.71, *p* = 0.49, Figure 2G), the mean intra-bout pause duration (0.218 ± 0.012 and 0.202 ± 0.010; *t* = 1.18, *p* = 0.25, Figure 2H) and the mean hyper-bout pause duration (0.049 ± 0.002 and 0.053 ± 0.002; *t* = −1.31, *p* = 0.20, Figure 2I) were also not significantly different at the two time points.

The mean frequency of vocalizations was also consistent between the two time points (75.07 ± 1.59 and 74.60 ± 2.02; *t* = 0.20, *p* = 0.84, Figure 2J), as was the mean maximum jump size of vocalizations (8.81 ± 0.80 and 9.04 ± 1.19; *t* = −0.28, *p* = 0.78, Figure 2K). Furthermore, there was no difference in the mean log of the power of vocalizations (22.773 ± 0.452 and 21.321 ± 0.592; *t* = 1.69, *p* = 0.11, Figure 2L) between time points.

In summary, there were no significant differences between the means of the two recordings for any of the commonly analyzed USV features suggesting stability of these features across the two recordings.

### Individual Variability between the Recordings Taken 1 h Apart

**Figure 3:**
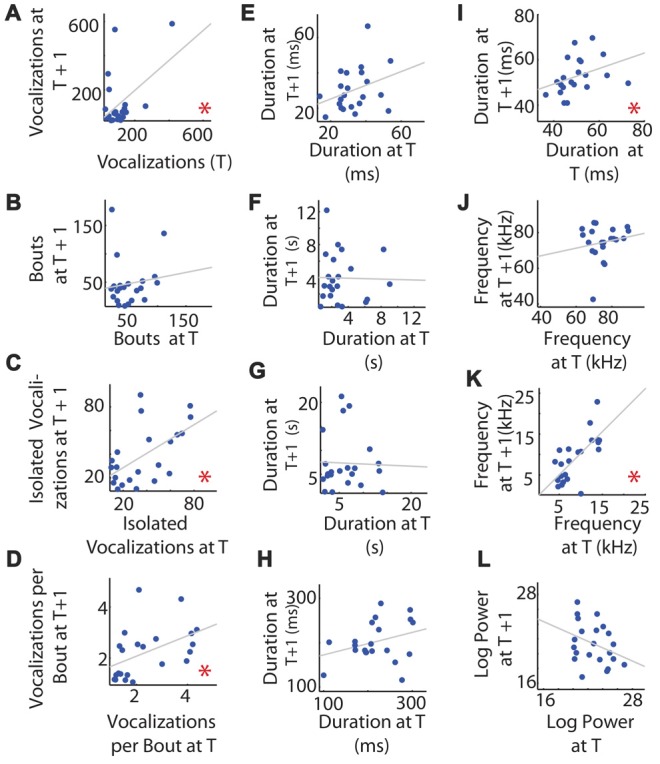
Consistency of mouse vocalization recordings separated by 1 h of rest in an individual. **(A)** Vocalization number. The number of vocalizations recorded at time *t* (on the *x*-axis) and *t* + 1 h (*y*-axis). Gray line shows the least squares regression. **(B)** Number of bouts per recording. **(C)** Number of isolated vocalizations. **(D)** Vocalizations per bout. **(E)** Mean vocalization duration. **(F)** Mean duration of pauses between vocalizations. **(G)** Mean pause duration of inter-bout vocalization. **(H)** Mean pause duration of intra-bout vocalization. **(I)** Mean pause duration of hyper-bout vocalization. **(J)** Mean frequency of vocalizations. **(K)** Mean pitch jump of vocalization. **(L)** Mean log power of vocalizations. ^*^Indicates a significant correlation.

We next looked at individual variability between the two recordings. Data are summarized in Table 1. We first looked to see if pups that were highly vocal at time *t* were also vocal at time *t* + 1, and found that pup vocalizations in the first and second recordings were significantly correlated (*R* = 0.472, *p* = 0.027, Figure 3A). The number of isolated vocalizations per recording (*R* = 0.567, *p* = 0.006, Figure 3C) and the number of vocalizations per bout (*R* = 0.472, *p* = 0.027, Figure 3D) were also significantly correlated. The number of bouts per recording was not correlated between the two time points (*R* = 0.131, *p* = 0.560, Figure 3B).

**Table 1:** Cohort 1 data comparing mouse vocalization parameters between the two recordings in C57BL/6J mice.

	*P* value	*t*-stat	*R*	*P*-value of *R*
Number of vocalizations	0.58	−0.56	0.59	**0.0042**
Number of bouts	0.90	0.13	0.16	0.47
Percent of isolated vocalizations	0.68	−0.42	0.57	**0.006**
Vocalizations per bout	0.68	0.42	0.47	**0.03**
Duration of whistles	0.96	0.05	0.27	0.22
Duration of pauses	0.32	−1.03	−0.02	0.94
Duration of inter-pauses	0.50	−0.68	−0.03	0.89
Duration of intra-pauses	0.31	1.05	0.30	0.19
Duration of hyper-pauses	0.11	1.67	0.52	**0.013**
Log of power	0.10	1.71	−0.34	0.12
Size of maximum frequency jump	0.93	−0.09	0.71	**0.0002**
Mean frequency	0.58	0.56	−0.05	0.82
Varaiblity of duration of vocalization	0.31	−1.03	−0.11	0.64
Varaiblity of duration of pauses	0.15	−1.51	−0.19	0.40
Varaiblity of mean frequency	0.19	−1.35	0.69	**0.0004**
Variability of maximum frequency jump	0.58	0.56	0.71	**0.0002**
Varaiblity of log of power	0.49	0.70	−0.08	0.73
Varability of vocalizations per bout	0.85	0.20	0.41	0.06

*The parameter correlations between the two time points are also shown. Significant data (*p* < 0.05) are shown in bold*.

With respect to the duration of vocalizations and pauses, surprisingly, neither were correlated between recording sessions for individuals (*R* = 0.329, *p* = 0.135, Figure 3E and *R* = −0.021, *p* = 0.926, Figure 3F, respectively), nor was the inter-pause duration (*R* = −0.032, *p* = 0.887, Figure 3G), intra-pause duration (*R* = 0.316, *p* = 0.163, Figure 3H), or hyper-pause duration (*R* = 0.365, *p* = 0.104, Figure 3I).

We next asked whether the features of the frequencies of the vocalizations were consistent between the two recordings for an individual, hypothesizing that this was likely as frequency can be determined by the shape and physical attributes of the larynx and related structures and is therefore likely to be mouse-specific. Surprisingly, the mean vocalization frequency measured in hertz was not significantly correlated between individuals across recording sessions (*R* = 0.170, *p* = 0.449, Figure 3J). However, the maximum frequency jump per vocalization was significantly correlated (*R* = 0.705, *p* = 0.00025, Figure 3K).

Due to their high energy, USVs do not transmit well and show directional specificity. We hypothesized that, due to this high degree of attenuation, vocalization power would be highly dependent on the direction the pup was holding its neck and would therefore not be individually consistent across recordings. Consistent with this hypothesis, the mean log of the power of the vocalization between time points for individuals was not significantly correlated (*R* = −0.340, *p* = 0.122, Figure 3L).

In summary, the individual number of vocalizations per unit time, percentage of isolated vocalizations, the vocalization number per bout, the hyper-pause duration, and the size of the maximum frequency jump were significantly correlated between the two recordings, while the bout number, duration of the whistles, duration of pauses (all pauses taken together, inter-pauses and intra-pauses), the log of power, and the mean frequency were not significantly correlated between the two recordings.

### Dispersion Index

**Figure 4:**
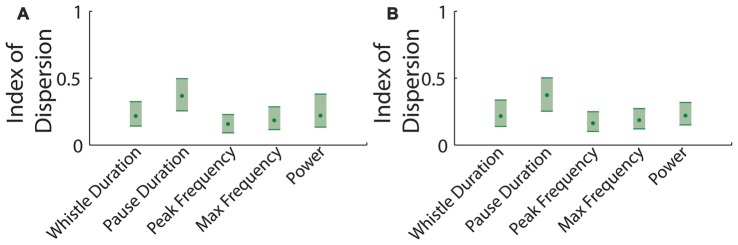
Dispersion index of parameters in mouse vocalization recordings. **(A)** Dispersion index for data taken at time *t*. Shaded errors represent 95% confidence intervals (CI). **(B)** Dispersion index for data taken after 1 h of rest.

We also examined which of these features showed the least variability. We calculated the dispersion index (the variance divided by the mean) for the whistle duration, pause duration, peak frequency of each vocalization and the maximum frequency jump per vocalization. Pauses in between vocalizations had a consistently higher dispersion index. This was perhaps not surprising, since there are at least two statistically distinct types of pauses (Figure 4; Ey et al., 2013; Barnes et al., 2016).

### Consistency of the Variability in Recordings

**Figure 5:**
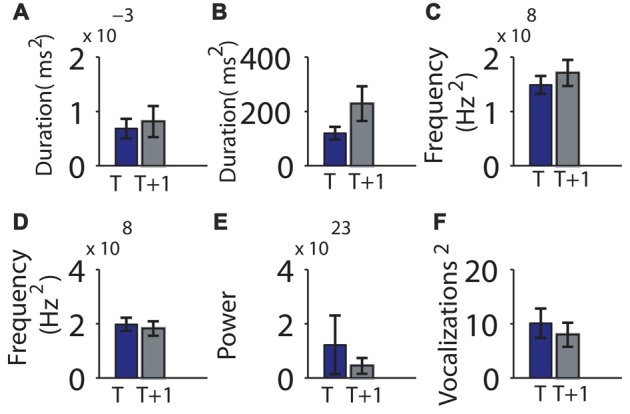
Consistency of the variability in mouse vocalization recordings separated by 1 h of rest in an individual was not significant. **(A)** Variability of vocalization duration at time *t* (on the *x*-axis) and *t* + 1 h (*y*-axis). **(B)** Variability of pause length. **(C)** Variability of the mean frequency of vocalizations. **(D)** Variability of mean pitch jump of vocalization. **(E)** Variability of power. **(F)** Variability of number of vocalizations contained in a bout. Error bars indicate the standard error of the mean.

We next examined the relative variability of the means between sessions, asking whether a parameter with low variability during the first session also had low variability during the second session. The group means of the variance of vocalization duration, pause duration, log of power, pitch frequency, or mean maximum frequency jump did not differ significantly between time points (Figure 5, Table 1). However, the *individual* variability across recordings for vocalization duration, pause duration and power were not significantly more correlated than by chance. However the variability in pitch frequency or mean maximum frequency jumps were more correlated in individuals than by chance (Table 1).

### Replication

**Figure 6:**
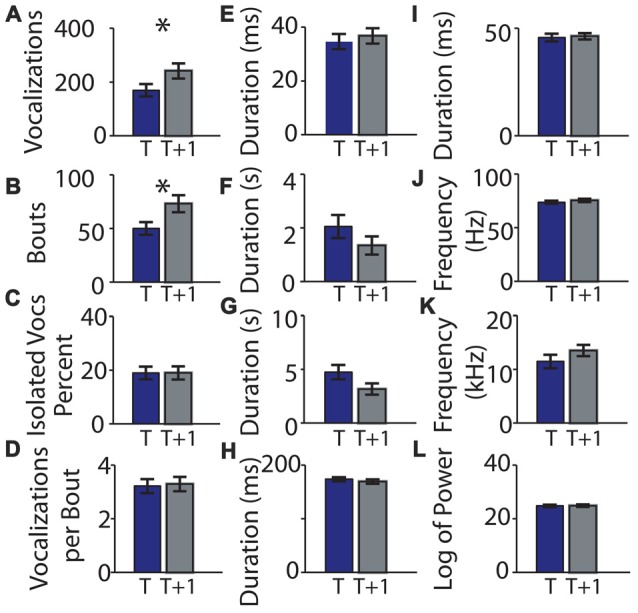
Replication of means of parameters of mouse vocalization recordings separated by 1 h is consistent. **(A)** Mean number of vocalizations in each recording. Blue bars indicate recording taken on post-natal day 5. Gray bars indicate recordings taken 1 h (*t* + 1 h of rest) later. Error bars are the standard error of the mean. **(B)** Number of bouts per recording. **(C)** Percentage of isolated vocalizations. **(D)** Vocalizations per bout. **(E)** Mean duration of vocalizations. **(F)** Mean duration of pauses between vocalizations. **(G)** Mean pause duration of inter-bout vocalization. **(H)** Mean pause duration of intra-bout vocalization. **(I)** Mean pause duration of hyper-bout vocalization. **(J)** Mean frequency of vocalizations. **(K)** Mean pitch jump of vocalization. **(L)** Mean log power of vocalizations. ^*^Indicates *p* < 0.05.

We next performed the same experiment with 28 different pups of the same age (P5) and strain (C57BL/6; cohort 2, summarized in Table 2). The experiment was conducted in a different laboratory by a different investigator. Seven pups were excluded for not having at least 20 calls in each recording. There was a significant difference in the number of syllables (*t* 0.324 ± 0.051 and *t* + 1 0.483 ± 0.062; *t* = −3.16, *p* = 0.00488, Figure 6A) and the bout number (0.259 ± 0.043 and 0.424 ± 0.057; *t* = −3.92, *p* = 0.00085, Figure 6B). There were no significant differences in the recordings taken at different time points for any of the other parameters (Figures 6C–L). Interestingly, although the cohort 2 strain was the same as that in cohort 1, many of the pup isolation call parameters differed significantly from cohort 1, even at baseline time *t*.

**Table 2:** Data from cohort 2 comparing parameters of mouse vocalizations between the two recordings in C57BL/6J mice.

	*P* value	*t*-stat	*R*	*P*-value of *R*
Number of vocalizations	**0.005**	−3.16	0.62	**0.003**
Number of bouts	**0.0009**	−3.92	0.67	**0.0009**
Percent of isolated vocalizations	0.999	0.002	0.38	0.09
Vocalizations per bout	0.77	−0.30	0.58	0.006
Duration of whistles	0.31	−1.05	0.75	**9 E-05**
Duration of pauses	0.17	1.42	0.18	0.44
Duration of inter-pauses	0.054	2.15	0.26	0.26
Duration of intra-pauses	0.10	1.70	0.76	**0.0001**
Duration of hyper-pauses	0.85	−0.19	−0.03	0.91
Log of power	0.92	−0.11	0.52	**0.01**
Size of maximum frequency jump	0.06	−1.98	0.62	**0.003**
Mean frequency	0.07	−1.88	0.81	**1 E-05**
Varaiblity of duration of vocalization	0.66	−0.45	0.34	0.14
Varaiblity of duration of pauses	0.26	1.16	−0.03	0.89
Varaiblity of mean frequency	0.95	0.07	0.36	0.10
Variability of maximum frequency jump	0.13	−1.58	0.44	**0.048**
Varaiblity of log of power	0.56	−0.60	0.83	**2 E-06**
Varability of vocalizations per bout	0.93	−0.08	0.44	**0.048**

*The parameters correlations between the two time points are also shown. Significant data (*p* < 0.05) are shown in bold*.

### Variability in a Different Strain

**Figure 7:**
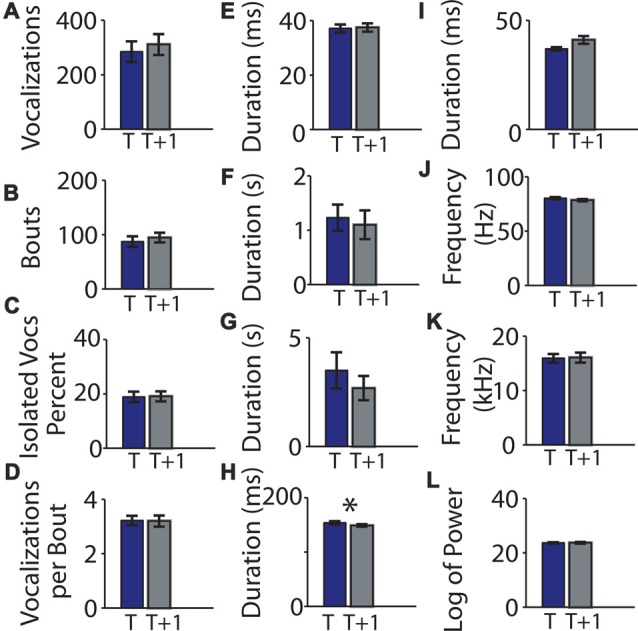
Means of parameters of mouse vocalization recordings from mice of genetically mixed background separated by 1 h are consistent. **(A)** Mean vocalization number in each recording. Blue bars indicate recording taken on post-natal day 5. Gray bars indicate recordings taken 1 h (*t* + 1 h of rest) later. Error bars are the standard error of the mean. **(B)** Number of bouts per recording. **(C)** Percentage of isolated vocalizations. **(D)** Vocalizations per bout. **(E)** Mean vocalization duration. **(F)** Mean duration of pauses between vocalizations. **(G)** Mean pause duration of inter-bout vocalization. **(H)** Mean pause duration of intra-bout vocalization. **(I)** Mean pause duration of hyper-bout vocalization. **(J)** Mean frequency of vocalizations. **(K)** Mean pitch jump of vocalization. **(L)** Mean log power of vocalizations. ^*^Indicates *p* < 0.05.

We next examined whether pups from a genetically mixed background would show the same high level of consistency between recording sessions using 27 C57BL/6J-129S5/SvEvBrd mice (cohort 3) under the same experimental protocol (Table 3). None of the examined features differed significantly between the two recording sessions (Figures 7A–L) except for intra-bout pauses (0.154 ± 0.003 and 0.149 ± 0.002; *t* = 2.64, *p* = 0.014, Figure 7H).

**Table 3:** Data from cohort 3 comparing mouse vocalization parameters between the two recordings.

	*P* value	*t*-stat	*R*	*P*-value of *R*
Number of vocalizations	0.38	−0.90	0.70	**4 E-05**
Number of bouts	0.26	−1.14	0.77	**3 E-06**
Percent of isolated vocalizations	0.92	−0.10	0.30	0.12
Vocalizations per bout	0.92	0.10	0.36	0.06
Duration of whistles	0.78	−0.29	0.75	**6 E-06**
Duration of pauses	0.66	0.45	0.33	0.10
Duration of inter-pauses	0.33	0.98	0.34	0.08
Duration of intra-pauses	**0.01**	2.64	0.82	**1 E-07**
Duration of hyper-pauses	0.08	−1.92	−0.09	0.76
Log of power	1.00	0.00	0.51	**0.01**
Size of maximum frequency jump	0.89	−0.14	0.53	**0.005**
Mean frequency	0.06	1.98	0.60	**0.001**
Variability of duration of vocalization	0.67	0.42	0.36	0.06
Variability of duration of pauses	0.56	0.59	0.10	0.64
Variability of mean frequency	0.38	0.90	0.45	**0.02**
Variability of maximum frequency jump	0.81	0.25	0.25	0.21
Variability of log of power	0.42	−0.82	−0.06	0.75
Variability of vocalizations per bout	0.21	1.28	0.15	0.44

*The parameter correlations between the two time points are also shown. Significant data (*p* < 0.05) are shown in bold*.

### Inference of Recording Identity

Supporting the findings that there were no significant differences between the two time points, LDA using recording features (vocalization number, percent of isolated vocalizations, mean vocalization duration, mean duration of pauses between vocalizations, mean vocalization frequency, mean maximum jump size of vocalizations, mean log of power of vocalizations) followed by nearest-neighbor classification did not classify recordings into those at time *t* or time *t* + 1 better than by chance (59.0% correctly classified, 95% CI, with shuffled data 34.0%–65.9%).

We used similar methods to ascertain whether an individual could be identified across recording sessions. Using parameters significantly correlated in individuals over the two time periods (number of vocalizations, percent of isolated syllables) and a one nearest-neighbor approach, an individual was correctly identified 7.1% of the time (95% CI 0%–9.5%).

## Discussion

Here we examined the individual and group variability of mouse pup vocalizations at two time points and in two separate experiments and strains. The group means of most mouse pup isolation call parameters were the same in recordings separated by 1 h of rest. Not only were the means consistent, but also the mean variability. These results support the use of these parameters as useful and repeatable measures in experiments using USVs to examine the effect of an intervention.

We also examined whether specific vocalization parameters in individuals were correlated between the two recording sessions, i.e., whether a mouse pup exhibiting a high number of vocalizations relative to the others during the first session repeated the behavior in the second session. The features seen in the first and second recordings were correlated for the vocalization number, percentage of isolated syllables, vocalizations per bout, duration of hyper-pauses, size of maximum frequency jump, and mean variability of maximum frequency jump but not for any of the other parameters (number of bouts, whistle duration, pause duration, inter-pause and intra-pause duration, log of power, mean frequency, variability of duration of vocalizations, variability of duration of pauses, variability of log of power, and variability of vocalizations per bout).

Two related parameters were significantly different in the replication experiment: number of vocalizations per recording and number of bouts per recording. Given that 18 different parameters were examined at three separate times, two significant (*p* < 0.05) differences are unlikely to be of great biological significance. In support of this, there were no significant differences in the third set of recordings in a different strain. However, the difference in the baseline values of these parameters highlight the importance of maintaining consistency in investigator and laboratory in these experiments.

These data can be compared with that of Rieger and Dougherty (2016) who found that many features of pup USV show consistent patterns within a recording session, but inconsistent patterns across postnatal development. Rieger and Dougherty (2016) data supported the conclusion that pup USV is most strongly influenced by “state”-like variables. We found that minimizing effects of maturation, by recording twice within a day, many features of mouse vocalizations were correlated for individuals, suggesting that different rates of development might add to variability across days.

A limitation of our data set is that the vocalizations in the second set of recordings may be affected by disruptions caused by the first set of recordings. Indeed, Hofer et al. (1994) has found that, in rats, the number of pup isolation calls can be doubled or even tripled by a brief exposure to the dam either in the testing chamber or elsewhere. In rats this effect, called maternal potentiation, can be seen following 30 min of contact with the dam (Hofer et al., 1994, 1998). This potentiation, after a brief 5 min reunion with dam and siblings, has been shown to occur in 8–12 day old mouse pups as well (Scattoni et al., 2008, 2009). In this experiment, it is possible that the consistency of the pup USVs is affected by maternal potentiation. However, the number of USVs calls did not increase between the two time points except in cohort 2. This does not rule out the possibility that other aspects of the calls were affected by the previous recording.

Mice from a genetically mixed background also showed no significant differences in the means of the parameters tested across the two time points with the exception of intra-bout pauses. Pause length had the highest index of dispersion, supporting the idea that hyper-bout, intra-bout and inter-bout pauses represent inherently different subpopulations of pauses (Ey et al., 2013; Barnes et al., 2016).

In conclusion, mouse vocalization parameters are extremely stable across recording sessions. These data provide evidence that the means of these USV features can be regarded as consistent in assays that should nevertheless control for maturation effects.

## Ethics Statement

Study was approved by the Animal Studies Committee at Washington University in St. Louis (IACUC equivalent), Washington University protocol 20130179 and 20160167.

## Author Contributions

TDB designed experiments, performed experiments, analyzed data and wrote the article. MAR designed experiments, performed experiments and edited the article. JDD designed experiments and edited the article. TEH designed experiments, analyzed data and edited the article.

## Conflict of Interest Statement

The authors declare that the research was conducted in the absence of any commercial or financial relationships that could be construed as a potential conflict of interest.
